# Carbon quantum dots composite for enhanced selective detection of dopamine with organic electrochemical transistors

**DOI:** 10.1007/s00604-024-06722-5

**Published:** 2024-10-01

**Authors:** Jillian Gamboa, Reem el Attar, Damien Thuau, Francesc Estrany, Mamatimin Abbas, Juan Torras

**Affiliations:** 1 Departament d’Enginyeria Química, EEBE, Universitat Politècnica de Catalunya, Av. Eduard Maristany, 10-14, 08019 Barcelona, Spain; 2 Barcelona Research Centre in Multiscale Science and Engineering, Universitat Politècnica de Catalunya, Av. Eduard Maristany, 10-14, 08019 Barcelona, Spain; 3 Univ. Bordeaux, CNRS, Bordeaux INP, IMS, UMR 5218, Pessac, 33607 France

**Keywords:** Carbon quantum dots, Poly(3, 4-ethylenedioxythiophene), Organic electrochemical transistor, Bioelectronics, Dopamine, Differential pulse voltammetry; Amperometry

## Abstract

A compact organic electrochemical transistors (OECT) sensor enriched with carbon quantum dots (CQDs) was developed to enhance the transconductance of an electropolymerized poly(3,4-ethylenedioxythiophene) (PEDOT) film, enabling the precise and selective detection of dopamine (DA). Accurate monitoring of DA levels is critical for diagnosing and managing related conditions. Incorporating CQDs, we have achieved a remarkable up to threefold increase in current at the DA detection peak in differential pulse voltammetry. This enhancement showcases superior selectivity even in the presence of high concentrations of interferents like uric acid and ascorbic acid. This material significantly boosts the sensitivity of OECTs for DA detection, delivering an amperometric response with a detection limit of 55 nM and a broader detection range (1 − 500 µM). Our results underscore the potential of low-dimensional carbonaceous materials in creating cost-effective, high-sensitivity devices for detecting DA and other biomolecules. This breakthrough sets the stage for the development of next-generation biosensors for point-of-care diagnostics.

**Graphical Abstract:**

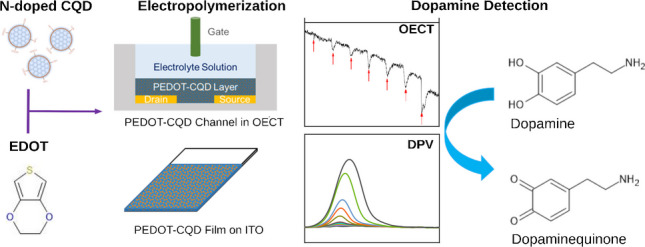

**Supplementary Information:**

The online version contains supplementary material available at 10.1007/s00604-024-06722-5.

## Introduction

Since their accidental discovery in 2004 during the separation and purification process of single-walled carbon nanotubes (SWCNT) by Xu et al. [1], carbon quantum dots (CQDs) have attracted increasing attention due to their unique structure and properties. These compounds exhibit a complex carbogenic structure, often comprising a mixture of crystalline structures reminiscent of graphene nanolayers combined with amorphous carbon. Predominantly spherical in shape, CQDs feature a crystalline nucleus composed of a blend of carbons, primarily graphitic (sp^2^ hybridization) or arranged in graphene sheets fused with diamond-like structures holding an sp^3^ hybridization, alongside amorphous structural elements [2, 3]. An interesting aspect of CQDs is their abundance of polar groups on the surface, including carboxylic residues, which vary depending on the synthetic route employed. These groups confer excellent solubility in water and serve as anchoring points for subsequent functionalization with other desired species or enhance affinity towards specific substances in detection processes [2].

Thanks to their interesting properties, CQDs have been utilized across a broad spectrum of fields, including chemical sensors, biosensors, bioimaging, nanomedicine, catalysis, and energy [4, 5]. Particularly in electrochemical applications, CQDs exhibit characteristics such as electronic conductivity, electron transfer sites, high surface area, and photoactive reaction centers, which greatly enhance the performance of electrochemical sensors [6]. Indeed, their integration with various materials commonly employed in electrocatalysis and electrochemical sensing, such as graphene, carbon nanotubes, metal oxides, and conductive polymers, further enhances their electrochemical properties, providing new opportunities in the electrochemical detections of bioanalytes [6].

Dopamine (DA) is a crucial neurotransmitter involved in regulating movement, motivation, memory, and other functions. Dysfunction of dopaminergic neurons is linked to neurological disorders such as Parkinson’s disease, Alzheimer’s disease, bipolar disorder, restless leg syndrome, autism, and schizophrenia [7–9]. Accurate detection of DA levels in biological systems is essential for the diagnosis, treatment, and prognosis of these conditions. However, electrochemical detection of DA is challenging due to its low clinical concentrations. For example, DA levels in plasma and urine are in the nanomole and micromole ranges, respectively [10, 11]. Additionally, substances like uric acid and ascorbic acid, which have similar oxidation potentials and are present in body fluids at concentrations up to 500 times higher than DA, interfere with detection [12]. Developing methods for rapid and sensitive dopamine detection is vital for the routine analysis and diagnosis of neurological disorders and has garnered significant interest [12, 13].

The use of carbonaceous materials in electrochemical DA detection has been growing in recent years. These materials complement the advantages of electrochemical methods, such as fast response time, ease of use, low cost, and high miniaturization capacity [13]. Recent studies have shown how low-dimensional carbonaceous products can enhance the sensitivity of conductive polymers to DA [14]. For instance, Thondaiman et al. [15] utilized a Cu mesh coated with electropolymerized poly(3,4-ethylenedioxythiophene) (PEDOT) to support *B*,*N*-doped graphene quantum dots (*B*,*N*-GQD) for DA detection, showing excellent performance in interference tests and recovery of urine samples [16]. Similarly, a PEDOT surface doped with graphene quantum dots (GQD) exhibited high electrochemical performance for DA detection. Darroudi et al. [17] developed a neuronal probe based on a microelectrode array, utilizing multi-walled carbon nanotubes (MWCNT) and CQDs to decorate a PEDOT film coating on a Pt electrode, achieving high stability and sensitivity in monitoring DA.

Transistor-based sensors have been used to track the biological activity of various bioanalytes. For instance, artificial oligonucleotide receptors, known as aptamers, which can recognize specific targets with high specificity and selectivity, have been integrated into field-effect transistors to serve as molecular recognition elements for neurochemical sensors [18]. More recently, organic electrochemical transistors (OECTs) have emerged as advanced devices for tracking the biological activity of various bioanalytes. OECTs have been employed to monitor neuronal, ionic, and cellular activities, among other applications [19]. This technology has garnered significant interest due to its potential for creating highly sensitive and flexible biosensors for bioanalytes that undergo redox reactions, which can influence the gate electrode current of the transistor. Importantly, the detection capability of OECTs is not dependent on their size, making it feasible to produce miniaturized devices that remain both functional and sensitive. Additionally, OECTs possess intrinsic amplification capabilities that can enhance weak signals from low-concentration analytes, thereby improving the limits of detection (LOD). Furthermore, the flexible organic materials used in these devices facilitate their assembly on flexible substrates, promoting biocompatibility and integration with the soft tissues of the human body [20].

The recent incorporation of OECTs in DA detection has enabled the miniaturization of sensors for wearable devices [21], the development of nanometric needle-type OECTs for in situ dopamine monitoring [22], and even their integration onto flexible surfaces [23]. Many of these devices utilize conducting polymers like PEDOT:PSS, reinforced with electrochemical enhancers such as graphene or reduced graphene oxide [24, 25]. However, to our knowledge, the use of carbon quantum dots to enhance the electrochemical response of OECTs has not been widely applied in bioanalyte detection, beyond the use of inorganic CdS quantum dots for lysozyme detection [26].

**Fig. 1:**
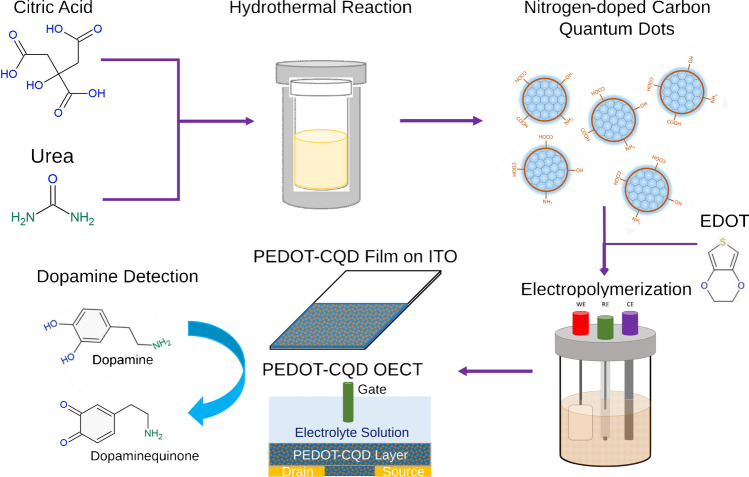
Schematic of PEDOT/CQD electrode and OECT fabrication for DA detection

The novelty of this work lies in the development of a miniaturized DA biosensor based on an OECT enhanced with CQDs to boost the electrochemical signal, aiming for future point-of-care applications. To achieve greater sensitivity, we developed and optimized an electropolymerized PEDOT film reinforced with CQDs on an indium-tin-oxide (ITO) substrate, using differential pulse voltammetry (DPV) (Fig. 1). This approach was inspired by our recent research on the sensitivity of nitrogen carbon quantum dots (NCQDs) to DA [14]. The chemical and morphological properties of the new carbonaceous material composite were confirmed through microscopy and spectroscopy analyses, followed by electrochemical characterization using electrochemical impedance spectroscopy (EIS). The new electroactive material’s effectiveness was demonstrated by fabricating a miniaturized OECT biosensor. This biosensor, calibrated at various DA concentrations, exhibited a wide response range, underscoring the potential of CQDs to enhance electrochemical signals and their suitability for integration into future compact sensor devices.

## Materials and methods

### Materials

3,4-Ethylenedioxythiophene (EDOT, 97%), dopamine hydrochloride (DA), uric acid (UA, 99%), citric acid (CA, 99%), ascorbic acid (AA, 99%), urea, phosphate buffer saline (PBS), indium-tin-oxide–coated polyethylene terephthalate film (ITO-PET), diethyl ether (98%), *N*,*N*-dimethylformamide (DMF, 99.9%), ethyl acetate, and lithium perchlorate (LiClO_4_, 95%). All these reagents were obtained from Sigma-Aldrich, Germany.

### Carbon quantum dots synthesis

Carbon quantum dots (CQDs) were fabricated via hydrothermal synthesis, as described by Paulo-Mirasol et al. [14] A total of 1.051 g of CA was mixed with 2 g of urea and 10 mL DMF. Once a clear homogeneous solution was obtained, it was carefully transferred into a 50-mL Teflon-lined autoclave reactor and subjected to heating at 160 °C for 16 h. The CQDs obtained were precipitated by utilizing a diethyl ether/ethyl acetate mixture (4:1 v/v) and subsequently centrifuged at 4500 rpm. The resulting precipitate was dried and securely stored in an airtight container at 4 °C.

### PEDOT and PEDOT/CQD fabrication

The electropolymerization process involved using 0.1 M LiClO_4_ as the supporting electrolyte and a 2.84 mg mL^−1^ (20 mM) EDOT solution in an aqueous media, performed both with and without CQDs on a 1 cm^2^ ITO-PET substrate with square shape. Various concentrations of CQDs were examined: 0.71, 1.42, and 2.84 mg mL^−1^, corresponding to EDOT:CQD (w/w) ratios of 4:1, 2:1, and 1:1, respectively. The electropolymerization mixture consisting of LiClO_4_ and EDOT with and without CQD was placed in an ultrasonic bath for 15 min to fully disperse the EDOT within the aqueous media. The ITO-PET substrate was submerged in the electropolymerization mixture together with an Ag|AgCl electrode as the reference and stainless steel as the counter electrode. Polymerization was conducted for 300 s at a constant potential of 1.1 V. These parameters led to a charge deposition of 300 mC cm^−2^.

### Physical–chemical characterization

The films underwent chemical characterizations using Fourier transform infrared spectroscopy (FTIR). The FTIR spectra were recorded using the Jasco 4100 spectrophotometer, with 64 scans per sample, spanning wavenumbers from 4000 to 600 cm^‒1^.

The UV–Vis absorption of CQDs solubilized in Milli-Q water was measured using the Shimadzu UV-3600, starting from the wavelength of 600 to 200 nm. A concentration of 0.125 mg mL^−1^ was used to obtain clear absorption peaks.

XPS analysis was conducted utilizing the SPECS XPS system, equipped with an XR50 Al anode operating at 10 kV and 150 W, MCD-9 electron detection, and the Phoibus 150 hemispherical analyzer. Data analysis was performed using Casa XPS software, with the carbon C1s peak binding energy set at 284.8 eV for spectrum calibration.

Morphological characterization of the films was performed using scanning electron microscopy (SEM). A Focus Ion Beam Zeiss Neon 40 instrument (Carl Zeiss, Germany) with an energy-dispersive X-ray (EDX) spectroscopy system was used. The SEM was conducted at 5 kV using the Inlens detector at various magnifications.

Topographic analysis of the film surface was analyzed using atomic force microscopy (AFM). The AFM images were obtained using an AFM Dimension microscope (Bruker) with a NanoScope IV controller and a silicon TAP 150-G probe (Budget Sensors, Bulgaria) at a force constant of 5 N m^−1^ and frequency of 150 kHz in tapping mode. The tip motion speed was set to 10 mm s^−1^ with a row scanning frequency of 1 Hz and scanning window of 10 × 10 µm^2^ and 2 × 2 µm^2^. To quantify the roughness of the films, three sections across the height channel were taken. The roughness values were quantified using *R*_max_, *R*_z_, and *R*_a_ (see definition in supporting information).

Transmission electron microscopy (TEM) was performed for the morphological analysis of CQDs. The TEM analysis was conducted using the J2010F TEM microscope, which is equipped with a field emission gun. CQDs dispersed in Milli-Q water at a concentration of 0.125 mg mL^−1^ were first sonicated then loaded and dried onto a carbon-coated microgrid. The size of the nanoparticles was calculated using the ImageJ software.

Electrochemical characterization was carried out using a conventional three-electrode cell and an Autolab potentiostat, PGSTAT. The setup included a Pt electrode as the counter-electrode (CE), Ag|AgCl (KCl 3 M) as the reference electrode, and a 0.01 M PBS solution as the supporting electrolyte. Electrical impedance spectroscopy (EIS) measurements were acquired at the open circuit potential (OCP), over a frequency range of 10^5^ to 0.1 Hz at room temperature. Cyclic voltammetry (CV) was performed with an initial and final potential of − 0.1 V, and a reverse potential of + 0.8 V, employing a scan rate of 100 mV s^−1^.

### Dopamine detection via differential pulse voltammetry (DPV)

DPV measurements were conducted with a step size of 0.015 V, a modulation amplitude of 0.05 V, a modulation time of 5 s, and an interval time of 10 s, spanning the potential range from − 0.1 to + 0.8 V. All experiments were conducted in a 0.01 M PBS solution at pH 7.4, using a Pt electrode as the counter electrode (CE) and Ag|AgCl (KCl 3 M) as the reference electrode. To prevent DA polymerization, a stock solution was prepared in 0.01 M PBS, with the pH adjusted to 4. Increasing volumes of this DA stock solution were sequentially introduced and analyzed via DPV to establish the calibration curve, covering the concentration range from 0.25 to 500.0 µM.

The same experimental setup was used to investigate the effect of increasing concentrations of uric acid and ascorbic acid on a constant dopamine concentration. In these tests, dopamine was first added to the PBS solution, followed by the sequential addition of either ascorbic acid or uric acid.

### Dopamine detection via organic electrochemical transistor (OECT)

The electrodeposition process employed an optimized EDOT:CQD ratio of 4:1 (w/w) to fabricate the PEDOT-CQD film on the channel of an OECT. The OECT configuration comprised a glass substrate, gold electrodes for the source and drain, and a SU8 insulating layer (Fig. 1). Additionally, a glass ring was positioned over the channels that served as a reaction chamber for the DA detection. The ring was filled with 1 mL of the electrodeposition solution (0.1 M LiClO_4_, 2.84 mg mL^−1^ EDOT, and 0.71 mg mL^−1^ CQDs) as the supporting electrolyte, while a platinum electrode and a pseudo silver wire reference electrode were dipped in the solution for use as counter and reference electrodes, respectively. The source and drain electrodes were used as the working electrode, and the three electrodes were connected to two different potentiostats. The first potentiostat was used to perform the electropolymerization by cyclic voltammetry at a rate of 50 mV s^−1^ between − 0.1 and + 1.1 V. The deposition begins at each of the source and drain electrodes. As the electropolymerization continues, the layer of PEDOT-CQD continues to grow and eventually bridge the source and drain electrodes. The second potentiostat applies a 10 mV potential across the source and drain electrodes, measures the current across them, and indicates whether a successful connection between the source and drain has been achieved [27]. This was performed until a channel current of 50 µA was achieved.

The electrical characteristics of the OECT were performed using the Keithley 4200 semiconductor analyzer. The electrolyte solution used was 0.01 M PBS at pH 7.4 and an Ag|AgCl electrode was used for the gate electrode. Transfer curves were measured to characterize the OECT. DA detection was performed by applying a constant drain voltage (*V*_d_) of − 0.4 V and a gate voltage (*V*_g_) of − 0.1 V. DA was sequentially added to the electrolyte solution every 500 s and the resulting change in the drain current (*I*_d_) was recorded.

## Results and discussion

Two electrodes were fabricated for DA detection and then thoroughly characterized and compared: one with pristine PEDOT and the other with nitrogen-rich CQDs added during electropolymerization, as shown in Fig. 1. Details on the fabrication, characterization, and CQD content optimization are in the Supplementary Information and Figs. S1 and S2.

### Electrode characterization

#### Chemical characterization

**Fig. 2:**
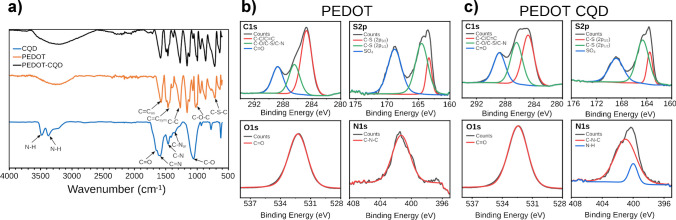
**a** FTIR spectra of PEDOT and PEDOT-CQD supported on an ITO-PET surface; high-resolution XPS spectra of C1s, S2p, O1s, and N1s for **b** PEDOT and **c** PEDOT-CQD electrodes

FTIR analysis was conducted to characterize the thin films of PEDOT, CQD, and PEDOT-CQD (Fig. 2), supported on an ITO-PET surface. The FTIR spectrum of the nitrogen-doped CQD exhibited similarities to prior studies [14, 28], revealing two distinct absorption bands at 3480 cm^−1^ and 3375 cm^−1^, attributed to N–H stretching vibrations typically observed in primary amines [29]. A prominent band at 1590 cm^−1^, with a shoulder at 1650 cm^−1^, originated from C = N and C = O stretching modes, respectively [28]. Additionally, the peak at 1460 cm^−1^ corresponded to a C–N stretching vibration [29], while peaks at 1360 cm^−1^ and 1089 cm^−1^ were associated with the stretching modes of aromatic C–N and C–O vibrations, respectively [30].

On the other hand, the bands at 1580 cm^−1^ and 1500 cm^−1^ in the PEDOT film were attributed to the asymmetric and symmetric stretching of the C = C bond in the thiophene ring. The peak at 1300 cm^−1^ corresponded to the C–C bond of the thiophene ring, and peaks at 1160 cm^−1^ and 1030 cm^−1^ were assigned to the stretching vibration bands of the ethylenedioxy group and the C–O–C groups, respectively. The contribution of C–S–C deformation was evident in the two peaks at 950 cm^−1^ and 713 cm^−1^ [31, 32].

Given the similarity of characteristic peaks between CQD and PEDOT, and the low concentration of CQD within the PEDOT-CQD film, the detection of CQD within the PEDOT film via FTIR was not feasible. Consequently, a more sensitive method (XPS) was employed to confirm the presence of CQD within the film. As depicted in Fig. S3, the main atomic components including C, S, O, N, In, and Sn are clearly identifiable in their respective XPS spectra. The last two elements are perfectly traceable due to the characteristic energy-binding peaks corresponding to an ITO film [33]. To elucidate the bonding types of the PEDOT and PEDOT-CQD components, high-resolution XPS spectra of C, S, O, and N were deconvoluted, as illustrated in Fig. 2b and c. The concentration of each component is listed in Table S1.

The high-resolution XPS spectra of C1s revealed three distinct energy-binding peaks (Fig. 2): C–C/C = C (284.4 eV), C–O/C–S (285.9 eV), and C = O (288.6 eV). Similarly, the PEDOT-CQD electrode exhibited comparable peaks, with a notable increase in the concentration of the C = O peak at 288.5 eV (Table S1), attributed to the carbon atom of the amide moiety [34]. In the S2p XPS spectra (Fig. 2), peaks at 163.5 and 164.6 eV correspond to S atoms in the PEDOT thiophene ring structure, appearing as spin-splitting double peaks S2p_3/2_ and S2p_1/2_, respectively [35]. The peak at 168.8 eV is attributed to sulfate impurities, which are observed on all surfaces, including the pristine ITO surface, as depicted in Fig. S4a. In addition to the S 2p region, the XPS spectra in the Cl 2p region were also analyzed. The Cl 2p spectra reveal characteristic spin-split peaks of perchlorate at 207.4 eV (Cl 2p3/2) and 208.3 eV (Cl 2p1/2), serving as a counterion to PEDOT (Fig. S3b) [35].

Additionally, a peak located at 531.8 eV indicated C = O, associated with the carbonyl of an ester group [36]. Furthermore, the high-resolution XPS spectra of N 1 s in the PEDOT film show a peak at 401.3 eV, which is assigned to the C–N–C group. In the PEDOT-CQD doped film, this peak becomes more pronounced and can be resolved into two contributions: N–H at 400.0 eV and C–N–C at 401 eV. This indicates an increase in the pyrrolic N type due to the presence of nitrogen-rich CQDs (Fig. 2b and c) [37, 38]. As summarized in Table S1, the relative elemental compositions of C = O and amide nitrogen in the PEDOT-CQD electrode were higher than those in the PEDOT electrode, while the remaining atomic compositions were similar or lower due to the reduction of PEDOT content in the final electrode composite. This was anticipated, considering that the CQDs used are N-doped during hydrothermal synthesis in the presence of urea. It is important to note that the nitrogen concentration detected here is expected to be relatively low, as the concentration of CQD within the film is low. Nonetheless, the increase in nitrogen and carbonyl moiety contents confirms the presence of N-CQDs within the film.

#### Morphological characterization

The SEM microscopy technique was employed to observe the morphology of both PEDOT and PEDOT-CQD films at two distinct polymerization times. Figure S5 illustrates the topographic images after 30 and 300 s of electropolymerization. Across all images, a consistent granular surface was observed and characterized by increasing granular density with longer electropolymerization time. Notably, the PEDOT-CQD film exhibited a notably higher granulation density compared to pristine PEDOT, regardless of electropolymerization duration. This phenomenon suggests a potential enhancement in PEDOT polymerization facilitated by CQDs. It is anticipated that the small CQD nanoparticles act as nucleation sites for EDOT electropolymerization owing to their favorable electrochemical properties, consequently leading to a greater number and size of PEDOT surface agglomerates. The presence of surface granules in the PEDOT-CQD film could be also attributed to CQD aggregate formation, as nanoparticles tend to aggregate naturally [39].

**Fig. 3:**
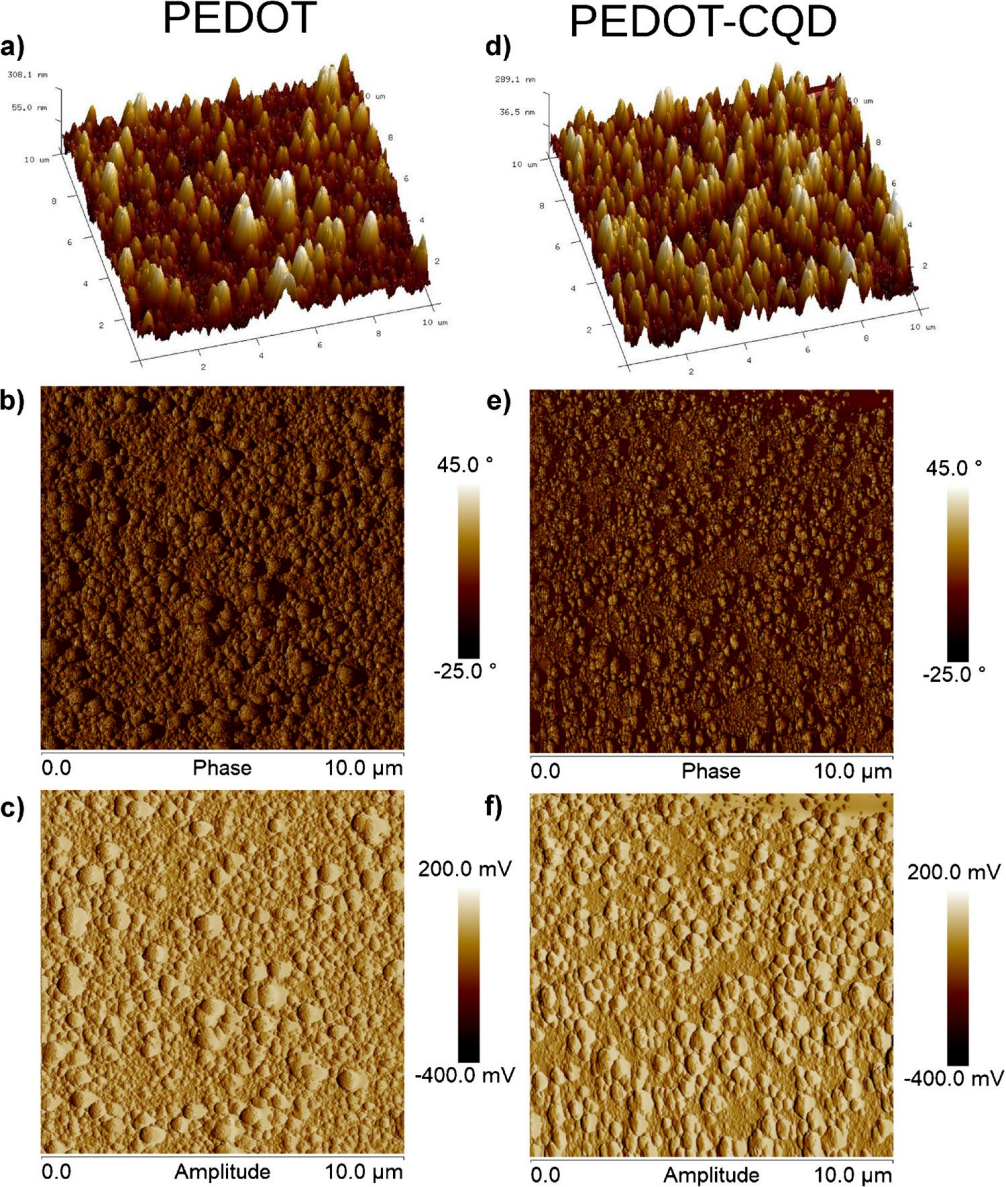
Illustrative AFM topography images (10 × 10 µm^2^) showcasing the morphology of **a**, **b**, **c** PEDOT films and **c**, **d**, **e** PEDOT-CQD films. The height (**a**, **d**), phase (**b**, **e**), and amplitude (**c**, **f**) channels are shown. The films were synthesized via electropolymerization over a 30-s duration

Further morphological analyses were conducted on the two films using AFM, as depicted in Fig. 3. In the AFM morphological explorations, only samples subjected to 30 s of electropolymerization were utilized. This decision was made because samples with prolonged electropolymerization times resulted in surfaces that were excessively rugged, potentially surpassing the exploration gradient limit in the Z direction.

Consistent with previous SEM observations, the AFM micrographs reveal a granular surface texture. Notably, the PEDOT-CQD samples exhibit a considerably rougher surface compared to pristine PEDOT. The roughness parameters listed in Table S2 corroborate these findings, with the PEDOT-CQD film displaying significantly higher roughness (*R*_z_ = 178.8 ± 12.8 nm) compared to PEDOT (*R*_z_ = 82.7 ± 8.7 nm). These results indicate that the addition of CQDs influences the ultimate conformational structure of the PEDOT film, resulting in a greater prevalence of larger granular aggregates. The increase in aggregate size enhances the film’s conductivity, a phenomenon linked to the reduction in boundary surfaces within a given volume or area as aggregate size increases, consequently lowering energy barriers to conduction [40]. This trend may offer further experimental support for the observed enhancement in the electroactivity of PEDOT films upon PEDOT-CQD doping, in conjunction with the intrinsic electroactivity contributed by the added CQDs themselves.

#### Electrochemical characterization

The electroactivity of both PEDOT and PEDOT-CQD electrodes supported on an ITO-PET substrate was characterized using CV and EIS in a three-electrode system. Here, an Ag|AgCl electrode served as the reference, and Pt acted as the counter electrode, all within a 0.01 M PBS solution as electrolyte.

CV measurements were conducted by scanning a potential window from − 0.1 to + 0.8 V against an Ag|AgCl electrode at a scan rate of 0.1 V s^−1^. Figure S6 depicts the CV results obtained at both electrodes in cycles 3 and 50. The specific capacitance (SC) of the PEDOT and PEDOT-CQD samples in cycle 3 was found to be 5.24 ± 0.63 mF cm^−2^ and 5.92 ± 1.07 mF cm^−2^, respectively, indicating a slight enhancement with the incorporation of CQDs. Regarding stability, the SC calculated in the redox cycle 50 was 5.03 ± 0.55 mF cm^−2^ and 6.22 ± 1.14 mF cm^−2^, respectively. Notably, the stability of PEDOT-CQD surpasses that reported in prior research where CQDs were directly deposited onto an ITO-PET electrode [14]. The function of CQDs as nucleation centers for EDOT polymerization in the formation of the PEDOT-CQD electrode not only avoids a significant loss of electroactivity but also leads to an improvement in electroactivity (*LEA* =  − 4.88%) after 50 CV cycles.

**Fig. 4:**
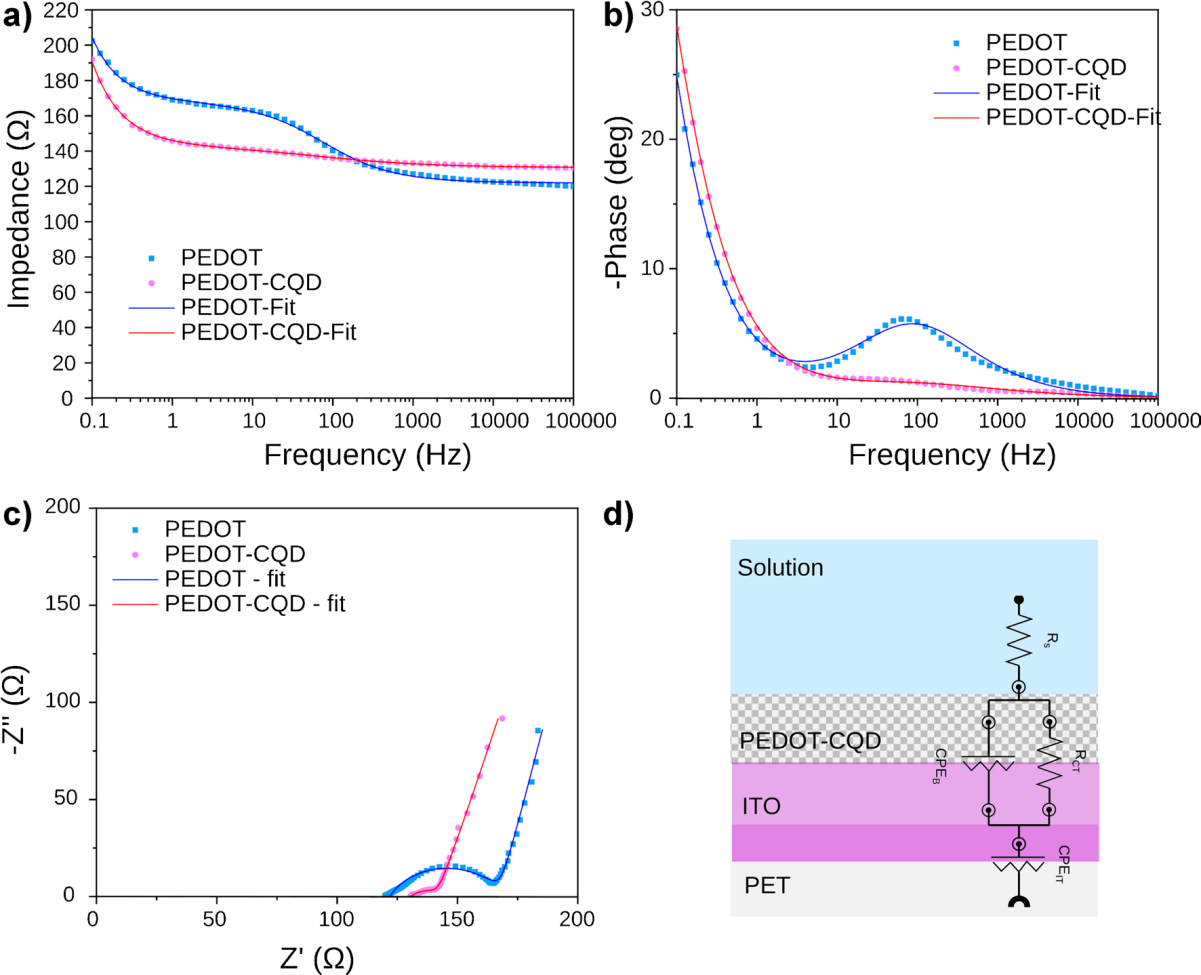
Measured EIS spectra for PEDOT (blue squares) and PEDOT-CQD (pink dots) electrodes: **a** impedance Bode plot, **b** phase Bode plot, and **c** Nyquist plot; **d** electrical equivalent circuit (EEC) model applied to fit the experimental impedance output (continuous line curves)

EIS measurements were conducted to analyze changes in the resistance and capacitance of the PEDOT electrode following polymerization in the presence of CQDs, forming the PEDOT-CQD system. EIS, a non-destructive technique, offers insight into various interfacial layers within an electrode [41] by assessing the impedance response under a sinusoidal potential across a frequency range from 10^−1^ to 10^5^ Hz. Figure 4 illustrates the results obtained for both PEDOT and PEDOT-CQD electrodes. In the impedance Bode plot (Fig. 4a), two distinct time constants were observed for the PEDOT electrode: one attributed to the PEDOT-ITO interface at higher frequencies and the other associated with the ITO oxide layer interfacing with the PET substrate at lower frequencies. Polymerization of PEDOT in the presence of CQD particles led to a reduction in |*Z*| at low frequencies. The differentiated behavior observed likely corresponds to the presence of PEDOT-CQD within the PEDOT polymerization.

Figure 4b illustrates the phase Bode plot, utilized for evaluating capacitive systems. Across the entire Bode plot, the phase angles remained below 30° at all frequencies, aligning with expectations for conductive materials. The PEDOT-CQD electrode exhibited higher conductivity compared to the PEDOT electrode, with the maximum on the phase Bode plot observed at about 10^2^ Hz disappearing.

The typical Nyquist plots of the as-prepared electrode reveal two parts; one is a semicircular shape for high and intermediated frequencies and the other is a relatively linear response at low frequencies. Nyquist plots are very useful to evaluate the electric double layer, electrode/electrolyte resistance, and charge transport in the electrode surface [42]. Comparing the Nyquist plot of both PEDOT and PEDOT-CQD electrodes (Fig. 4c), a nice semicircular shape at high-medium frequencies for the PEDOT can be observed, whereas PEDOT-CQD showed a more depressed and shorter semicircle shape, indicating a higher conductivity and a less ideal capacitor behavior of the double layer [43]. Both the real and imaginary components of the impedance decreased with the addition of CQDs to the PEDOT film. The impedance spectrum was modeled by a Randles electrical equivalent circuit (EEC). The model presents an electrolyte resistance (*R*_s_) in series with a parallel combination of a thin porous material resistance (*R*_ct_), called charge transfer resistance, and a constant phase element (CPE_B_), followed by another constant phase element (CPE_IT_) in series (Fig. 4d) [44]. The non-ideal capacitance element (CPE) is usually present as a result of the sample surface defects, such as porosity and surface roughness.

Table S3 outlines the constituents of the EEC employed to fit the EIS spectra data, achieving a chi-square (*χ*^*2*^) value within the range of 10^−3^ to 10^−4^, indicating a robust fit to experimental data. The Nyquist semicircle corresponds to the *R*_ct_ (charge transfer resistance) and CPE_B_ (constant phase element of the organic layer) elements observed at intermediate to high frequencies. A notable threefold reduction in resistance (*R*_ct_*)* was obtained upon incorporating CQDs into the PEDOT film, indicating enhanced electroactivity and facilitating charge transfer (measured at 11.9 Ω) from the organic detection layer to the ITO electrode. Furthermore, the PEDOT-CQD electrode exhibits a less ideal capacitive behavior of the system double layer compared to the PEDOT electrode, with *n* values of 0.52 and 0.71, respectively. A CPE element with an *n* value of 1 is associated with an ideal capacitor, while a value of 0 denotes a pure resistor. This deviation from ideal capacitive behavior in the PEDOT-CQD system may stem from the presence of CQDs within the bulk, leading to increased film heterogeneity and surface roughness. Conversely, the ITO layer demonstrates quasi-ideal capacitive behavior (CPE_IT_) with *n* values closer to unity (0.87 and 0.83 for the PEDOT and PEDOT-CQD systems, respectively), attributed to its smooth and homogeneous characteristics [45].

### Dopamine detection via differential pulse voltammetry (DPV)

DPV was employed to investigate the electrochemical response of both electrodes, namely PEDOT and PEDOT-CQD. DPV is known for its heightened sensitivity and superior resolution in DA detection compared to cyclic voltammetry (CV), making it a preferred analytical methodology in this context [46–48]. This method involves applying a series of voltage pulses to polarize the working electrode in a stair-steps shape slope of potential. The current difference measured immediately before each potential change of the pulse is used as a detection signal, significantly reducing the capacitive current contributions. This enables a better measurement of the faradic current associated with the analyte reaction, resulting in enhanced accuracy and sensitivity.

**Fig. 5:**
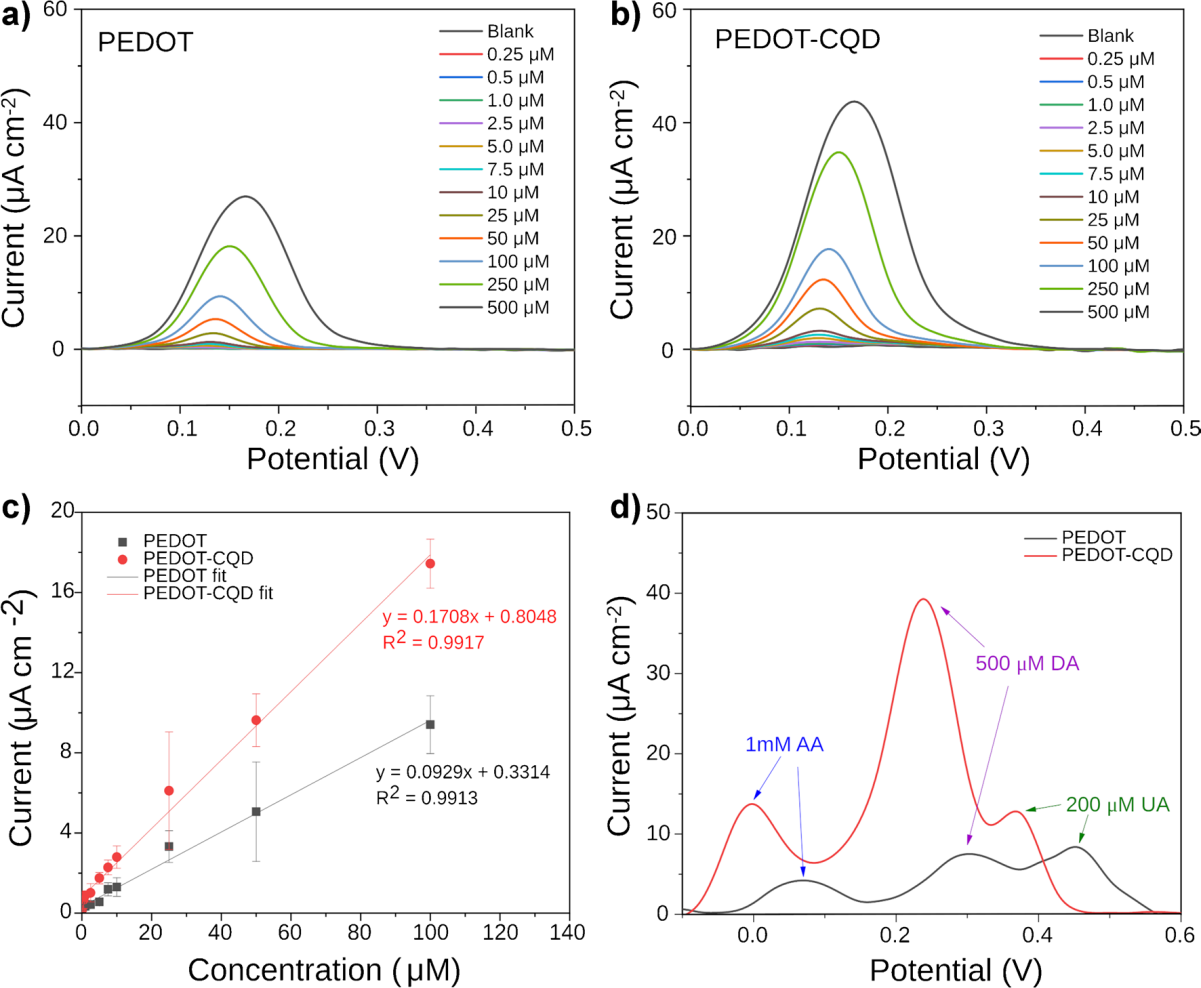
DPV measurements across a range of DA concentrations (0.25–500 µM) in PBS solution (0.01 M, pH 7.4) using both **a** PEDOT and **b** PEDOT-CQD electrodes; **c** linear calibration curves of DA at both electrodes; **d** selectivity test to assess DA detection in the presence of AA and UA

Figure 5a and b depict the DPV measurements across a range of concentrations from 0.25 to 500 µM of DA in PBS solution using both PEDOT and PEDOT-CQD electrodes, respectively. In the DPV detection of DA, the oxidation peak current observed at the PEDOT-CQD electrode significantly surpassed that recorded at the pristine PEDOT electrode. This outcome highlights the remarkable electrocatalytic activity exhibited by the PEDOT-CQD electrode towards the electrochemical reaction of DA, coupled with its exceptional reversibility and sensitivity. This heightened sensitivity is further illustrated by the calibration curves in Fig. 5c, demonstrating a twofold increase in sensitivity to DA using the CQD-doped samples (0.171 µA µM^−1^ cm^−2^) compared to PEDOT samples (0.093 µA µM^−1^ cm^−2^).

Several factors contribute to this enhanced sensitivity. Firstly, as indicated by the EIS results, CQD doping improves electroactivity, thereby facilitating charge transfer across the film during the DA redox reaction and resulting in increased current. Secondly, morphological data from both AFM and SEM reveal an increase in film roughness upon CQD doping, providing a larger surface area for DA oxidation and consequently amplifying the signal. Finally, the presence of amine and hydroxyl groups on the surface of the CQDs may form hydrogen bonds and/or electrostatic interactions with DA, promoting its interaction with the film surface and enhancing the likelihood of DA oxidation. The adsorption of dopamine on CQD has been the basis of several CQD-based sensors for dopamine via several techniques including fluorescence [49] and surface plasmon resonance [50].

Additionally, it is noteworthy that DPV analysis with PEDOT-CQD exhibits a lower LOD value (1.40 µM) compared to pristine PEDOT (5.53 µM), indicating a fourfold improvement in detection sensitivity.

Common interferents in DA detection, such as uric acid (UA) and ascorbic acid (AA), are electroactive compounds found alongside DA in human blood, typically with concentration ranges of 34–85 µM for AA and 120–450 µM for UA [51, 52]. Both of these biomolecules are capable of undergoing redox reactions, as the oxidation potentials of both UA and AA closely align with that of DA. Consequently, DPV selectivity was evaluated as a detection method for quantifying DA in a PBS solution containing a mixture of DA, AA, and UA across both electrodes. In Fig. S7, DPV measurements of both PEDOT and PEDOT-CQD electrodes at various DA concentrations (100–500 µM) are depicted in the presence of interferents at much higher concentrations typically found in human blood, including 200 µM AA and 200 µM UA. Notably, a distinct separation of peaks between AA, DA, and UA is observed in the CQD-doped electrode. Similarly, additional DPV experiments were conducted at a constant DA concentration while increasing the concentrations of UA and AA. The results indicated that the DA signal remains fairly constant, despite an increase in the signals from both UA and AA (Fig. S8). Furthermore, in Fig. 5d, the DPV measurements of both electrodes with a fixed concentration of DA (500 µM), AA (1000 µM), and UA (200 µM) demonstrate enhanced sensitivity and peak separation, particularly between UA and DA, facilitating better peaks distinction.

### Dopamine detection via organic electrochemical transistor (OECT)

In the past decade, organic electrochemical transistors (OECTs) have emerged as powerful tools in biosensing to detect different bioanalytes due to their high sensitivity, affordability, ease of fabrication, flexibility, and biocompatibility [53]. Among their applications, OECTs have been instrumental in detecting DA, leveraging their inherent amplification properties to push the limits of detection [54].

**Fig. 6:**
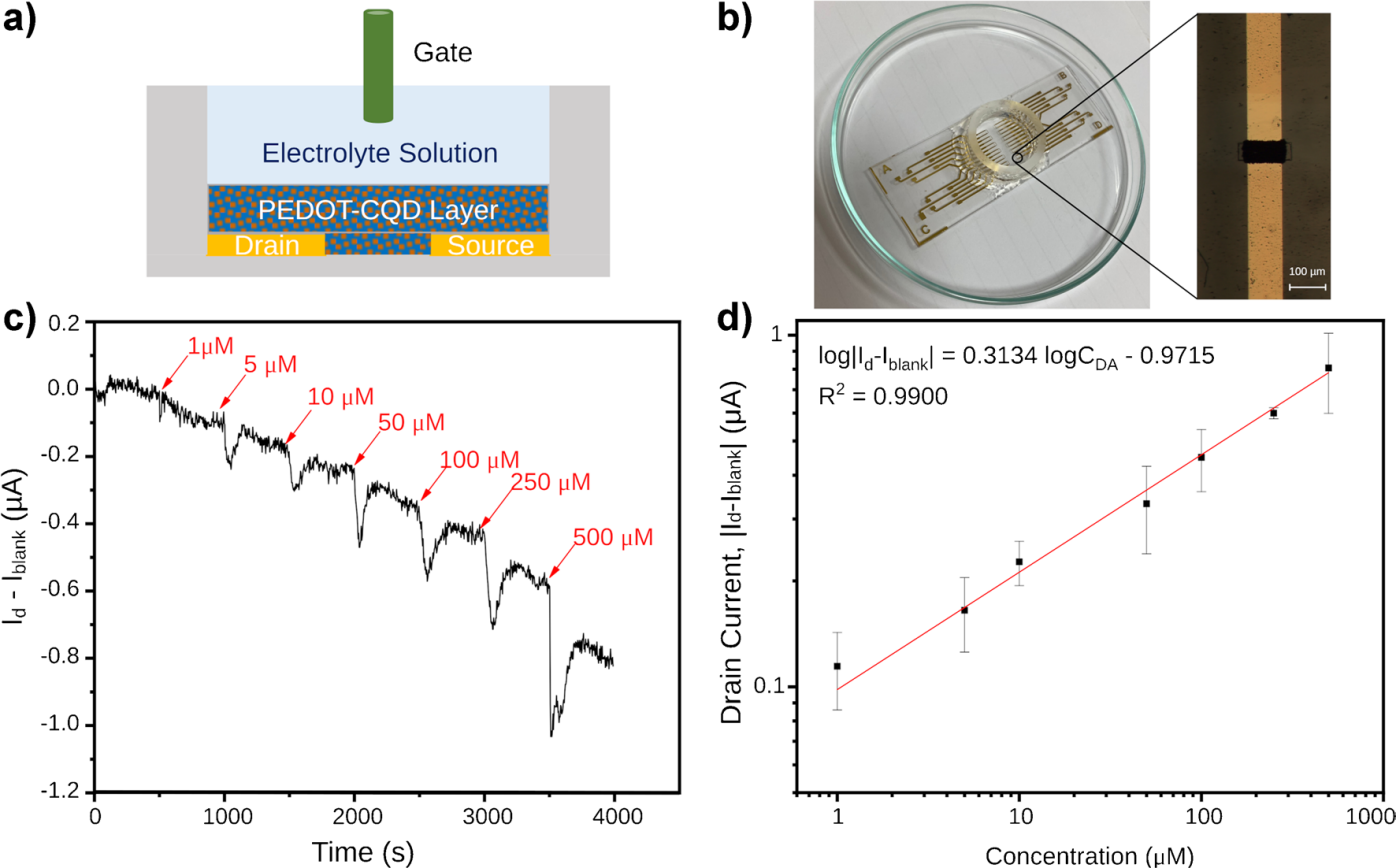
**a** Schematic of the PEDOT-CQD OECT device. **b** Optical image showcasing the OECT device. The inset reveals a microscope image detailing the 100-µm electropolymerized wide channel. **c** Amperometric response (*I*_d_ plotted with respect to time) of the PEDOT-CQD OECT recorded with incremental additions of DA. **d** Drain current response plotted with respect to DA concentration (*C*_DA_)

Building upon the characterization of the PEDOT-CQD electrode, a thin film of CQD-doped material was deposited by electropolymerization to serve as the channel material in a newly fabricated OECT. In a typical OECT setup, three electrodes—gate, source, and drain—are utilized (Fig. 6a), with the latter two connected by a channel, here made of PEDOT-CQD. Both the channel and gate electrode are immersed in an electrolyte solution, in this instance PBS (0.01 M, pH 7.4). A constant voltage bias (*V*_d_) is applied at the drain electrode, while the source electrode remains grounded. This applied *V*_d_ induces a current flow through the channel (*I*_d_), which can be modulated based on the potential applied at the gate electrode (*V*_g_). Figure 6b depicts the OECT device employed for DA detection. A layer of PEDOT-CQD film was electrodeposited via CV between the source and drain electrodes to assess the transconductance current (as shown in the inset of Fig. 6b).

Drain voltage modulates the current flow through the channel via electronic charge carriers, while gate voltage further modulates the current via ions in the electrolyte solution by changing the doping state of the channel material. To characterize OECTs, transfer curves were measured (Fig. S9a and b), which represent *I*_d_ when a *V*_d_ is applied across a cyclic sweep across a range of *V*_g_ values.

In the case of PEDOT-CQD films, the film is doped in its neutral state. A change in *V*_g_ towards more positive values leads to an injection of cations from the electrolyte towards the channel, resulting in the pairing of cations with ClO_4_^−^ (PEDOT dopant during electropolymerization), thereby dedoping the film and thus decreasing *I*_d_. This process is reversible; thus, when *V*_g_ is reversed, an increase in *I*_d_ is observed. Figure S9a and b depict the transfer curve of a PEDOT-CQD OECT, demonstrating slight hysteresis, as the doping and dedoping process is inherently imperfect, as expected.

DA engages in a reversible redox reaction, transitioning between DA and its oxidized form, dopamine-o-quinone. When DA is present in the electrolyte solution of an OECT, it undergoes oxidation at either the channel or the gate. Consequently, this leads to a depletion of charge carriers in the channel, resulting in an anticipated reduction in *I*_d_. All measurements were conducted under steady-state conditions to detect DA in solution. A *V*_g_ of − 0.1 V and a *V*_d_ of − 0.4 V were applied to the transistor, and the resulting *I*_d_ current was recorded. Once a stable signal was attained, additive volumes of DA were sequentially added to construct the calibration curve.

To assess the diffusion effect and any alteration of the medium upon adding different volumes of DA to the PEDOT-CQD OECT setup, a preliminary study was conducted. For this purpose, 25 µL of a DA stock solution was introduced into the electrolyte solution of the PEDOT-CQD OECT, achieving a final concentration of 500 µM DA. As depicted in Fig. S9c, following the addition, there was an initial sharp decrease in current, succeeded by a slight increase, eventually reaching a plateau. Notably, this plateau did not return to the initial *I*_d_ value prior to DA addition. Conversely, when an equal volume of PBS solution was added as a blank, an initial dip in the *I*_d_ current was observed, which subsequently recovered to the initial value of the blank. This indicates that part of the initial dip in *I*_d_ could be attributed to the expected alteration in the solution. However, the dip in *I*_d_ resulting from the addition of one volume of the blank solution was approximately an order of magnitude smaller compared to the dip observed with DA addition, despite the equal volume. This suggests that the *I*_d_ measurements for DA determination remain unaffected by volume additions once the plateau is reached with measurements taken 450 s after DA addition.

Figure 6c depicts the real-time change of *I*_d_-*I*_blank_ in a PEDOT-CQD OECT device, observed during the sequential additions of DA at varying concentrations every 500 s in a PBS solution. To construct the calibration curve, the |*I*_d_-*I*_blank_| change from the aforementioned plot was correlated with DA concentration, plotted on a logarithmic scale for optimal fitting (Fig. 6d). Electrochemical data are conventionally represented logarithmically for improved function fitting [22, 55, 56].

The device’s performance can be elucidated by determining the limit of detection (LoD), which signifies the lowest analyte concentration reliably distinguishable from the blank measurement. For the PEDOT-CQD OECT, the LoD was calculated as 0.055 µM. This value was derived by multiplying the standard deviation of the blank signal by 3 (signal-to-noise ratio, *S/N* = 3) and subsequently substituting it into the logarithmic equation to ascertain the corresponding concentration. The standard deviation of the blank was calculated based on 100 s of an already equilibrated OECT blank signal, which was recorded prior to the addition of the first DA.

Furthermore, the device demonstrated robust operational stability even after 60 and 67 days of storage under ambient conditions following PEDOT-CQD electrodeposition (see Fig. S9a and b). Despite a decrease in channel conductivity, likely attributed to material degradation, the OECT maintained its functionality and conductivity throughout the 2-month storage period. The variations in drain current percentage upon the addition of 500 µM DA after prolonged storage are detailed in Table S4. Notably, the PEDOT-CQD OECT consistently exhibited a stable response to the same concentration of DA, highlighting its reusability, stability, and repeatability.

Consistent with our previous DPV measurements, we compared the DA detection sensitivity of the PEDOT-based OECT with that of the CQD-PEDOT-based OECT. As shown in Figure S10, the CQD-PEDOT OECT exhibits a significantly enhanced sensitivity compared to the standard PEDOT OECT, with an improvement of nearly fourfold. This substantial increase in sensitivity highlights the superior performance of the CQD-PEDOT configuration due to the CQD addition.

The performance of the PEDOT-CQD material to be used as a sensor was benchmarked against other methods reported in the literature for DA detection. Table 1 presents a compilation of state-of-the-art electrochemical sensors utilizing DPV and the OECT approach for DA detection. It is noteworthy that, while the utilization of PEDOT-CQDs films yields comparable results in the DPV method, its integration into OECT devices extends the detection range significantly, covering up to 500 µM of DA while retaining exceptional sensitivity with detection limits in the tens of nanomolar range, as shown in Table 1, consistent with other OECT sensors. Interestingly, to note, a recent study by Liang et al. [57] developed an OECT-based aptasensor that achieved an extremely low detection limit of 0.5 fM and a detection range of up to 10 nM, albeit with a required incubation time of 10 min per sample.

**Table 1:** Summary of sensors for DA reported in literature based on DPV and OECT methodology for the determination of DA

Sensor	Method	Detection range (µM)	LOD (µM)	Ref
MIP-OPPy	DPV	0.01–0.1	0.005	[58]
GQD/GCE	DPV	0.4–100	0.05	[59]
GQD/MWCNT	DPV	0.25–250	0.095	[60]
ErGO/PEDOT:PSS (7:3)	DPV	3–33	0.4	[61]
PEDOT-CQD	DPV	0.25–100	1.4	This work
PEDOT:PSS	OECT	5–100	6.0	[47]
Nafion(1.0%)–rGO/Pt	OECT	0.01–1	0.005	[24]
o-MIP/Pt	OECT	0.003–100	0.034	[62]
NIPS/PEDOT:PSS	OECT	0.001–100	0.043	[63]
PEDOT-CQD	OECT	1–500	0.055	This work

*GCE* glassy carbon electrode, *GQD* graphene quantum dot, *CQD* carbon quantum dot, *DPV* differential pulse voltammetry, *MIP* molecularly imprinted, *MWCNT* multiwall carbon nanotubes, *NIPS* nonionic fluorosurfactants, *OECT* organic electrochemical transistor, *OPPy* over-oxidized polypyrrole, *rGO* reduced graphene-oxide

Electrochemical methods based on redox processes at the electrode, such as those presented in this work using DPV or OECT, enable rapid sample analysis without the need for incubation periods. OECT, in particular, offers greater potential for miniaturization and simplicity in detection, making it ideal for modern wearable devices. It achieves highly promising detection limits due to the system’s intrinsic amplification factor. Moreover, adding CQDs to the electrode improves sensitivity and selectivity, promising valuable potential for medical applications.

## Conclusions

We have developed a miniaturized CQD-enhanced OECT sensor to improve the transconductance of a PEDOT film for the effective and selective detection of DA. The incorporation of CQDs, optimized using DPV, has increased the current at the DA detection peak by up to threefold. Material optimization demonstrates enhanced selectivity even in the presence of high concentrations of common interferents such as UA and AA. This new material significantly improves the sensitivity of OECTs for electrochemical detection of DA. Our findings indicate that low-dimensional carbonaceous materials hold great promise for developing low-cost, high-sensitivity devices for detecting dopamine and other biomolecules. This advancement paves the way for next-generation compact biosensors for point-of-care diagnostics, offering robust and reliable tools for medical and healthcare applications.

## Supplementary Information

Below is the link to the electronic supplementary material.

Supplementary file1 (PDF 1735 KB)

## Data Availability

Data will be made available upon reasonable request.
