# Supplementary material for: Carbon quantum dots composite for enhanced selective detection of dopamine with organic electrochemical transistors
Source: Mikrochim Acta. 2024 Oct 1;191(10):639. doi: 10.1007/s00604-024-06722-5 (PMC11445364; doi:10.1007/s00604-024-06722-5)
Supplement: Supplementary file 1 — Supplementary file1 (PDF 1735 KB) [file 604_2024_6722_MOESM1_ESM.pdf]

Supporting Information

Carbon Quantum Dots Composite for Enhanced Selective Detection of Dopamine with Organic Electrochemical Transistors

Jillian Gamboa [1,2], Reem el Attar [3], Damien Thuau [3,], Francesc Estrany [1,2], Mamatimin Abbas [3], and Juan Torras [1,2,*]*

[1] Departament d'Enginyeria Química, EEBE, Universitat Politècnica de Catalunya. Av. Eduard Maristany, 10-14, Barcelona, 08019, Spain

[2] Barcelona Research Centre in Multiscale Science and Engineering, Universitat Politècnica de Catalunya. Av. Eduard Maristany, 10-14, Barcelona 08019, Spain

[3] Univ. Bordeaux, CNRS, Bordeaux INP, IMS, UMR 5218, Pessac 33607, France

*Corresponding authors: Damien Thuau (damien.thuau@ims-bordeaux.fr) and Juan Torras (joan.torras@upc.edu)

CQDs Characterization

The CQDs were further characterized using UV-VIS spectroscopy and TEM. Fig. S1 shows the UV-VIS spectrum of 0.125 mg mL^{-1} CQDs dissolved in Milli-Q water, revealing several shoulder bumps and absorption peaks. The maximum absorbance at 210 nm corresponds to the π-π* transition of sp^2 aromatic domains, while the bands at 340 nm and 410 nm represent the n-π* transitions of C=O/N=O groups. Additionally, the shoulders at 255 nm and 280 nm indicate π-π* transitions of C=C bonds [1, 2, 3]. On the other hand, TEM micrographs (Fig. S1b) reveal spherical nanoparticles with an average size of 2.07 ± 0.35 nm (Fig. S1c).

Electrode fabrication and CQD optimization

Two distinct types of electrodes were fabricated for DA detection, followed by comprehensive characterization and comparative analysis. Specifically, two different PEDOT films were synthesized through electropolymerization on an ITO-PET substrate. One film comprised the pristine polymer, while the other incorporated varying amounts of nitrogen-rich CQDs during the electropolymerization process. Previous research by the authors demonstrated the efficacy of nitrogen-rich CQDs in the determination of neurotransmitters such as DA [2]. Additionally, it has been noted in the literature that PEDOT electrodes exhibit successful performance in DA determination [4]. Considering the electrochemical enhancement capabilities conferred by CQDs to electroactive systems [5], it is anticipated that the synergistic effect of both electrochemically active materials will enhance the electrochemical properties of the hybrid material, thereby facilitating the detection of neurotransmitters such as DA. Consistent with the methodology proposed by Chen et al [6], nitrogen-rich CQDs were synthesized via the hydrothermal method and incorporated in varying proportions (i.e., EDOT:CQD ratios of 4:1, 2:1, and 1:1, respectively)

during the electropolymerization process to generate distinct electroactive film samples (refer to the materials and methods section). These samples were then compared through their utilization as electrodes for recording output electric current signals in a DPV analysis of a 0.5 mM DA solution (refer to Fig. S2). The results show a significantly enhanced DA detection peak current in electrodes incorporating CQDs. In fact, the CQD-infused electrodes exhibit an almost threefold increase in signal compared to pristine PEDOT, showcasing a notable enhancement in DA detection sensitivity achieved through PEDOT doping with CQDs. Moreover, electrodes with higher CQD concentrations (EDOT:CQD ratio of 2:1) show only marginal signal improvement. Conversely, when testing lower CQD concentrations (not shown, with an EDOT:CQD ratio of 8:1), the signal towards 0.5 mM DA improved relative to PEDOT, albeit less than that obtained with an EDOT:CQD ratio of 4:1. Consequently, based on these outcomes, the optimal EDOT:CQD ratio selected was 4:1. Henceforth, the EDOT:CQD ratio will remain 4:1 unless specified otherwise, and these electrodes will be referred to as PEDOT-CQD.

Table S1 XPS binding-energy peaks (***BE***, in eV) assignation and concentration (**Conc.**, in %) of PEDOT and PEDOT-CQD films spectra.

	**PEDOT**		**PEDOT-CQD**	
	BE	**Conc.**	***BE***	**Conc.**
C 1s	284.8	42.0	285.1	44.5
O 1s	532.2	42.1	532.4	41.1
N 1s	401.5	1.8	400.3	3.2
S 2p	163.5	3.9	163.7	4.8
Cl 2p	207.6	2.4	207.8	1.8
In 3d	445.3	7.2	445.6	4.0
Sn 3d	487.1	0.6	487.4	0.5

Table S2 Roughness parameters from the surface analysis of AFM topography images of PEDOT and PEDOT-CQD films.

	R_{max} **(nm)**[a]	R_z **(nm)**[b]	R_a **(nm)**[c]
PEDOT	175.9 ± 70.8	82.7 ± 8.7	46.5 ± 8.6
PEDOT-CQD	267.9 ± 53.4	178.8 ± 12.8	70.5 ± 11.1

[a] R_{max} is the difference between the highest and lowest points in a section relative to the center line; [b] R_z is the average differences between the 5 lowest and 5 highest points across a selected section relative to the center line; [c] *Ra* gives the average value of the roughness curve relative to the center line.

Table S3 Resistance (R, in Ω), constant phase elements (CPE, in F cm^{-2} s^{n-1}) and its chi-square goodness of fitting to the EEC displayed in Fig. 5d. The error associated to each element of the EEC is written in parentheses and expressed in percentage (%).

	R_s	R_{CT}	CPE_B	n_1	CPE_{IT}	n_2	χ^2
PEDOT	121.9	45.9	2.90E-04	0.71	0.020	0.87	0.0052
	(0.3)	(1.8)	(10.8)	(2.4)	(1.3)	(1.3)	
PEDOT-CQD	130.7	11.9	3.34E-04	0.52	0.016	0.83	0.0007
	(0.1)	(4.2)	(17.6)	(5.3)	(0.5)	(0.5)	

Table S4 Drain current response of PEDOT-CQD OECT after addition of 500 μM DA at day 0 and day 60. Two experiments were performed at day 0, hence experiment 1 and experiment 2.

**Day**	**$(I_d$-$I_{blank})/I_{blank}$, (%)**
0 (Experiment 1)	3.92
0 (Experiment 2)	2.99
60	4.33

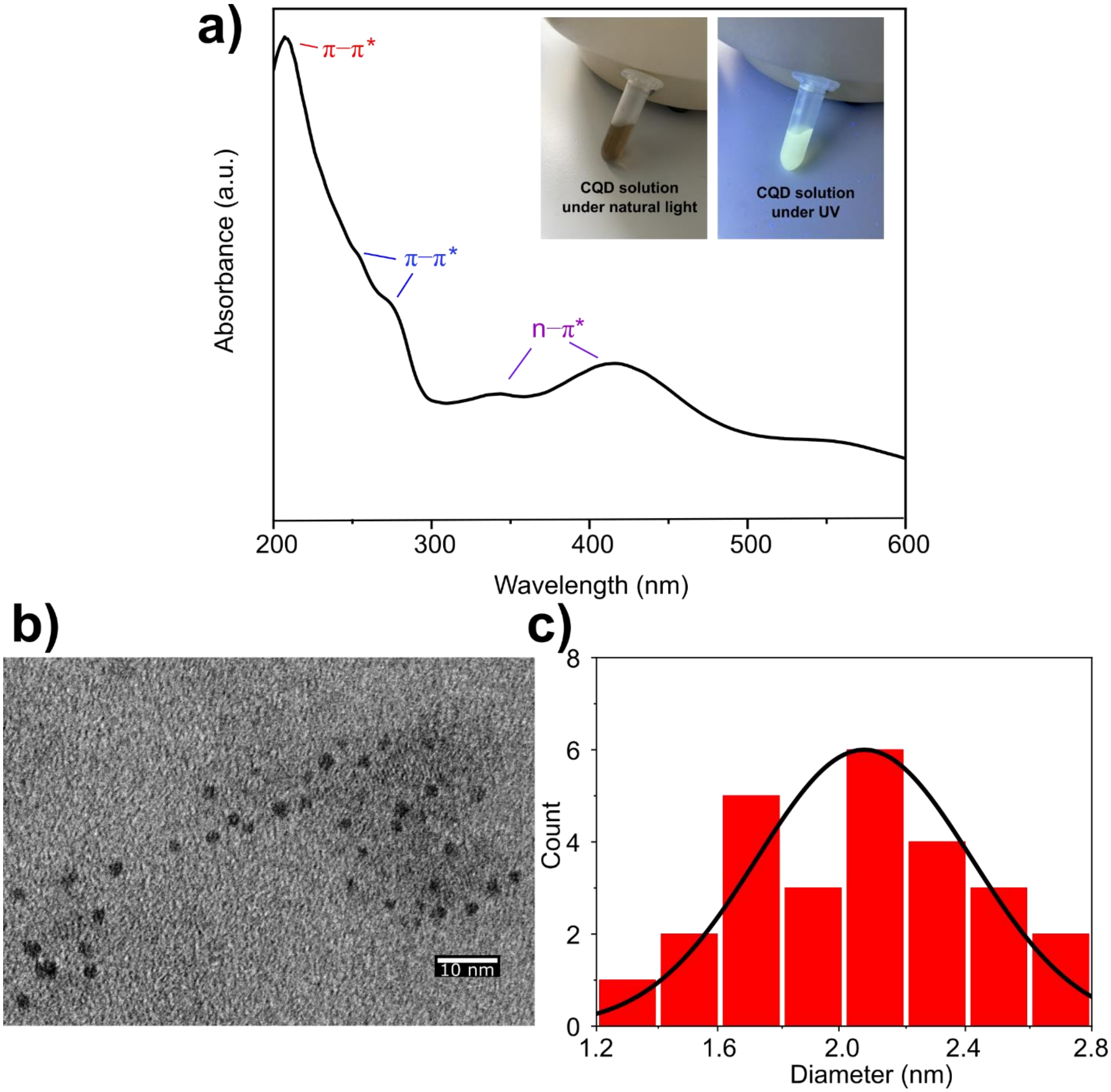

Fig. S1 Characterization of synthesized CQDs: (a) UV-VIS spectrum, (b) TEM micrograph, and (c) particle size distribution.

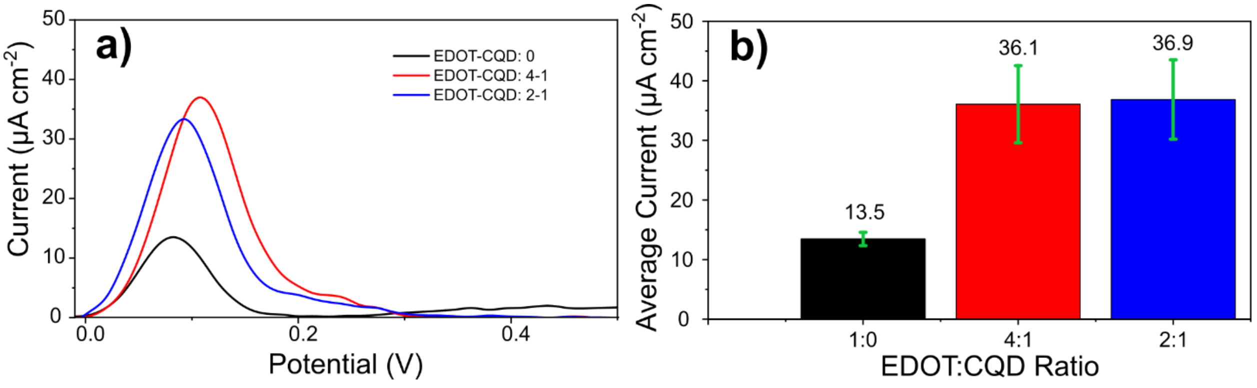

Fig. S2 a) DPV analysis on a 0.5 mM DA solution employing electrodes with various EDOT:CQD ratios, and b) comparing the resultant peak current values.

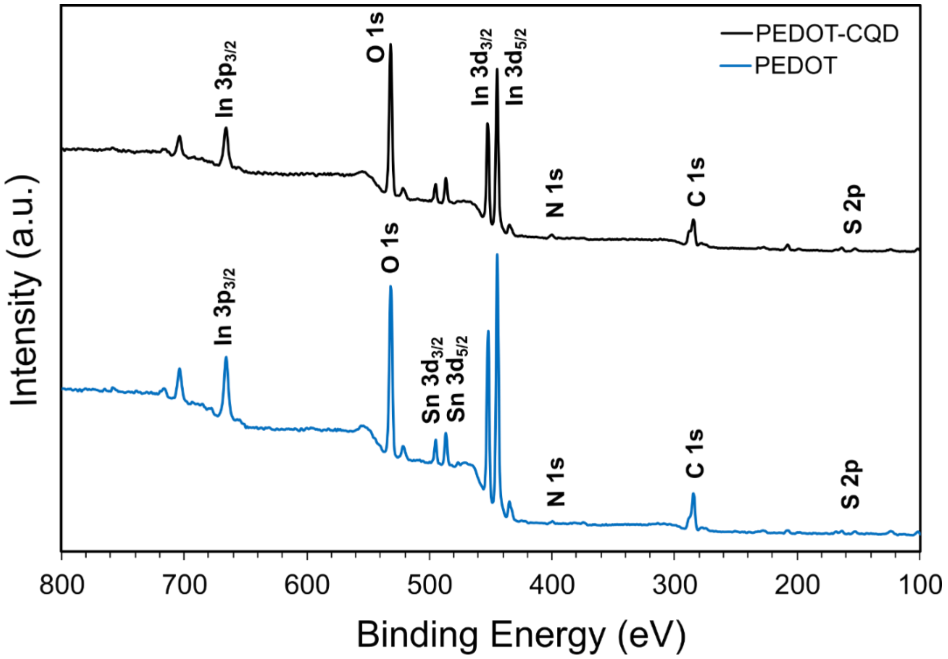

Fig. S3 XPS spectra of PEDOT and PEDOT-CQD electrodes.

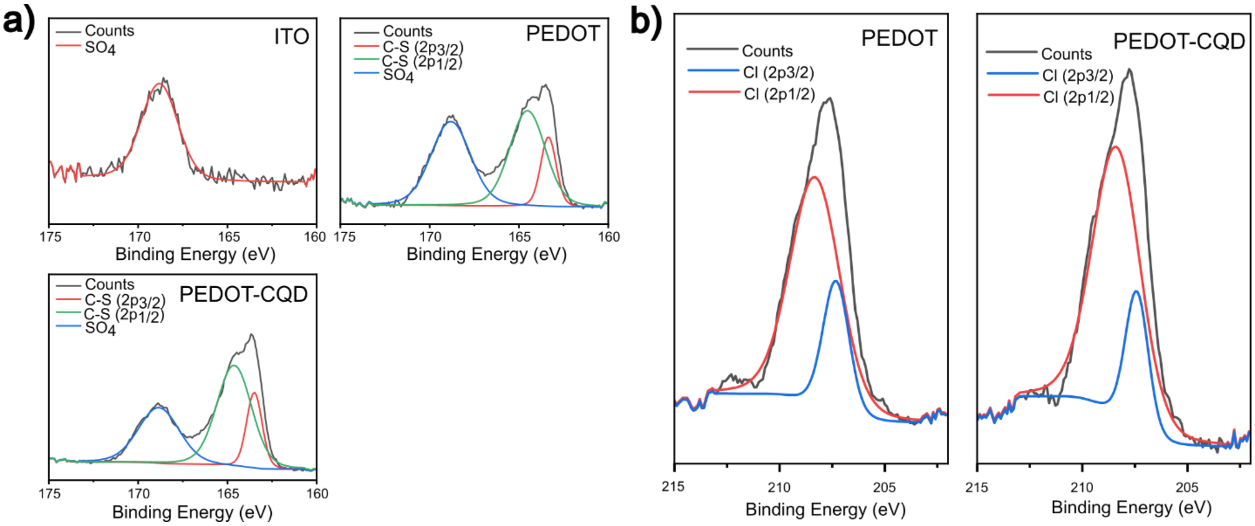

Fig. S4 High-resolution XPS spectra of (a) S2p for ITO-PET, PEDOT and PEDOT-CQD electrodes, and Cl2p for PEDOT and PEDOT-CQD electrodes.

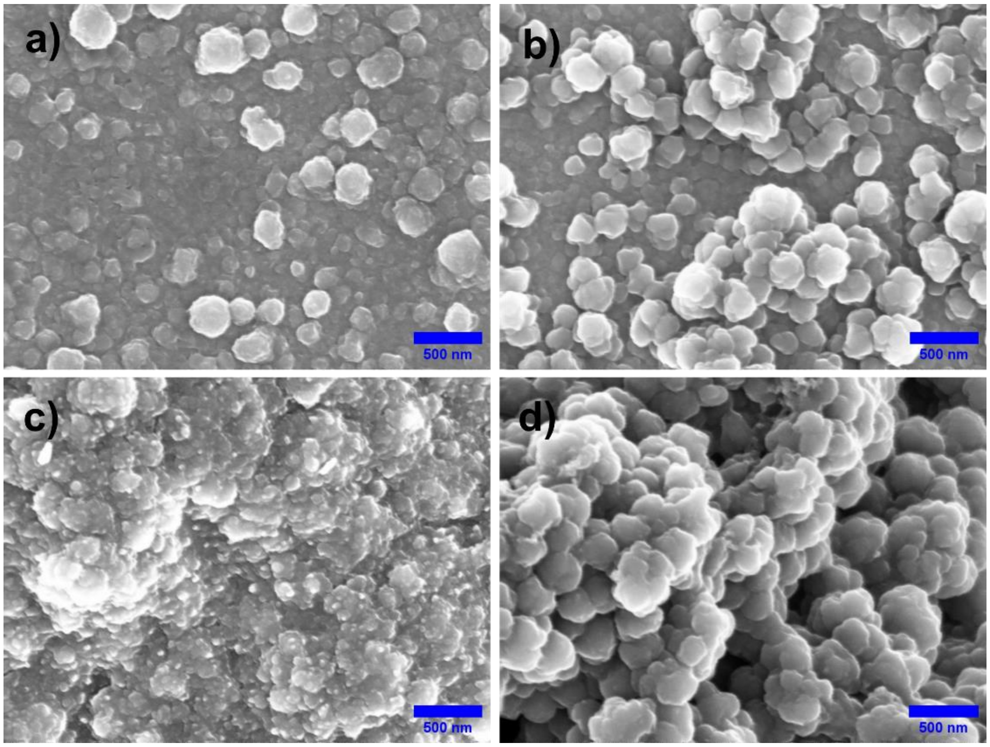

Fig. S5 SEM micrographs at 30,000x of magnification for PEDOT electropolymerized at a) 30 s and c) 300 s, and PEDOT-CQD electropolymerized at b) 30 s and d) 300 s.

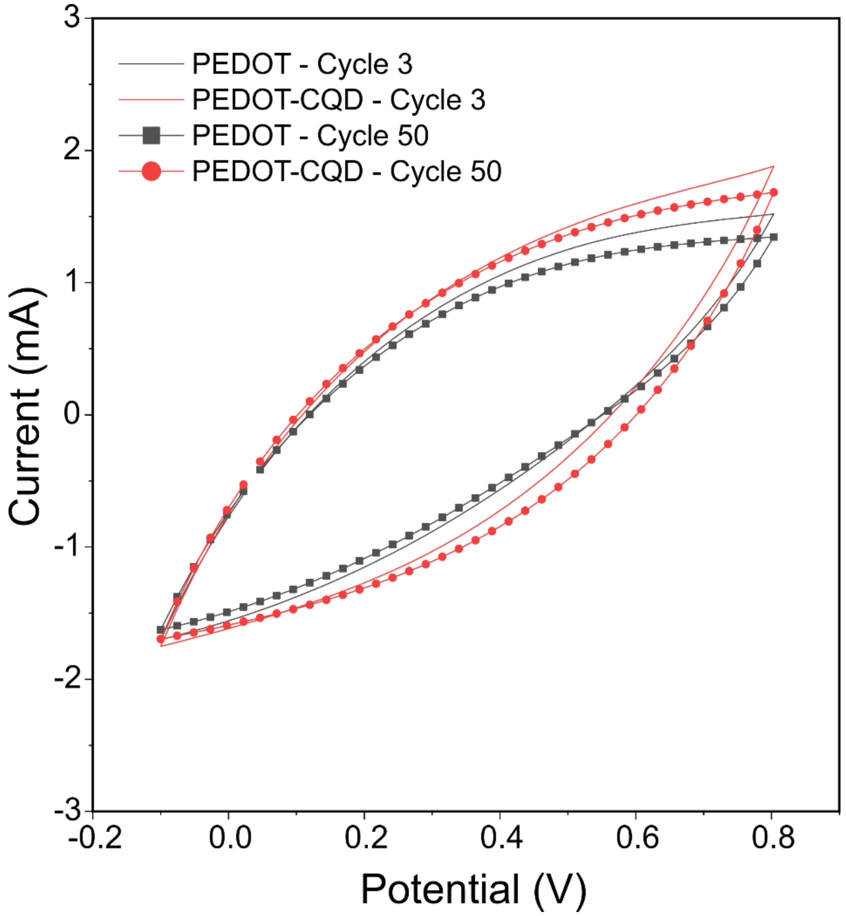

Fig. S6 Cyclic voltammograms of PEDOT and PEDOT-CQD films at the cycle 3 and 50. Voltammograms were obtained at the scan rate of 100 mV s−1 using 0.01 M PBS solution at pH 7.4.

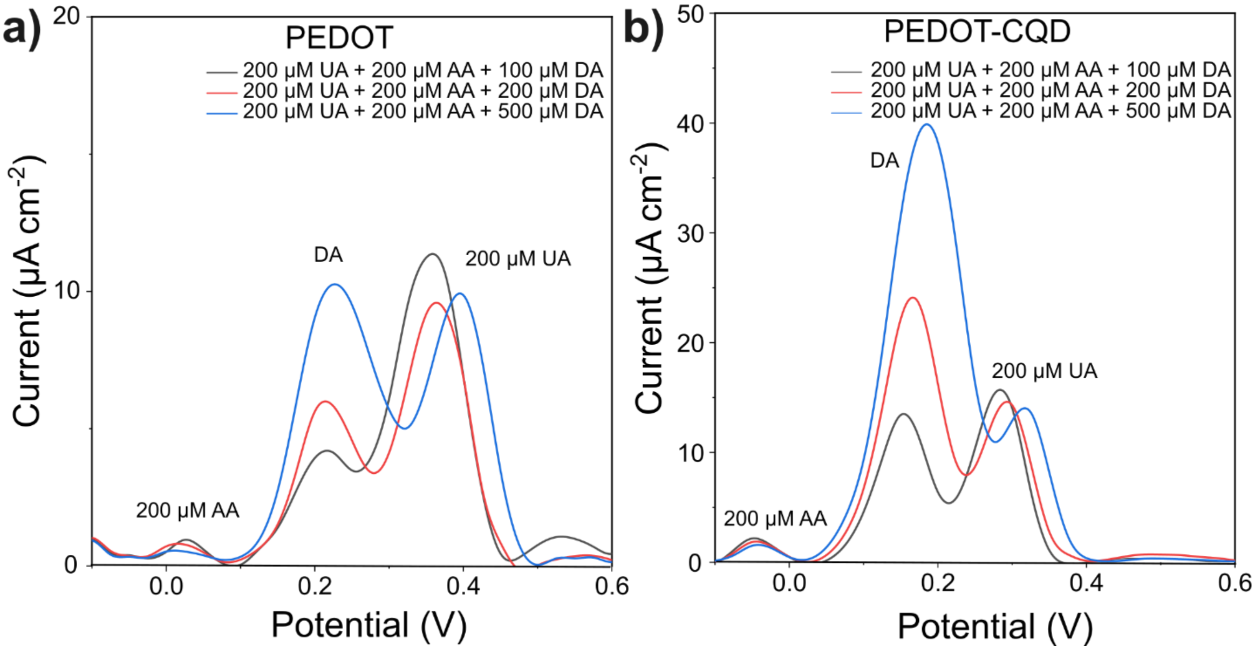

Fig. S7 DPV measurements at different DA concentration (100, 200 and 300 μM) in the presence of 200 μM UA and 200 μM AA using the (a) PEDOT and (b) PEDOT-CQD electrodes in PBS solution (0.01 M, pH 7.4).

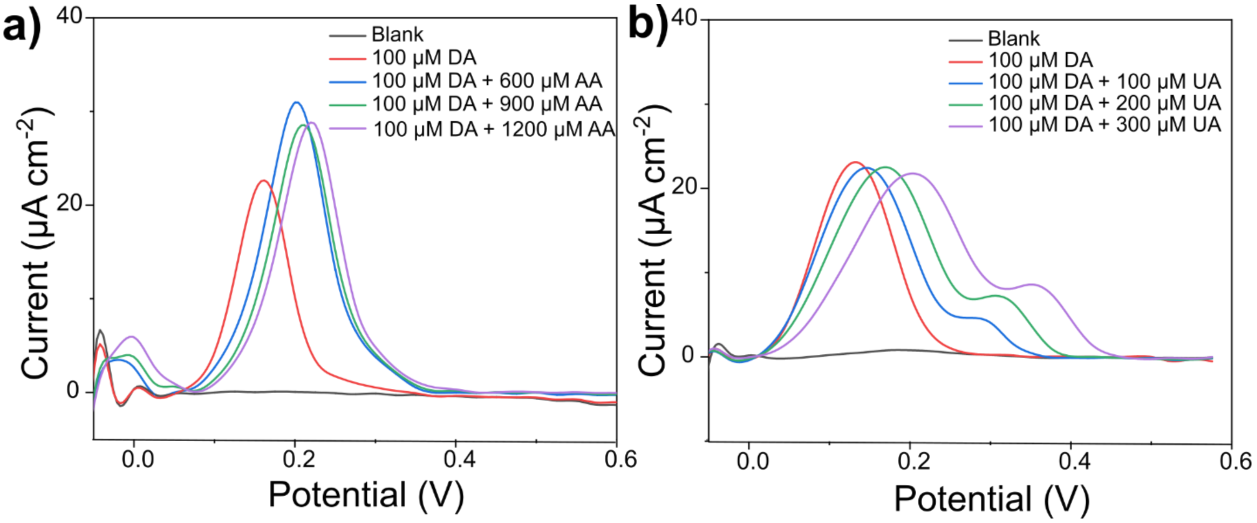

Fig. S8 DPV measurements at different a) AA and b) UA concentrations in the presence of 100 μM DA using the (a) PEDOT and (b) PEDOT-CQD electrodes in PBS solution (0.01 M, pH 7.4).

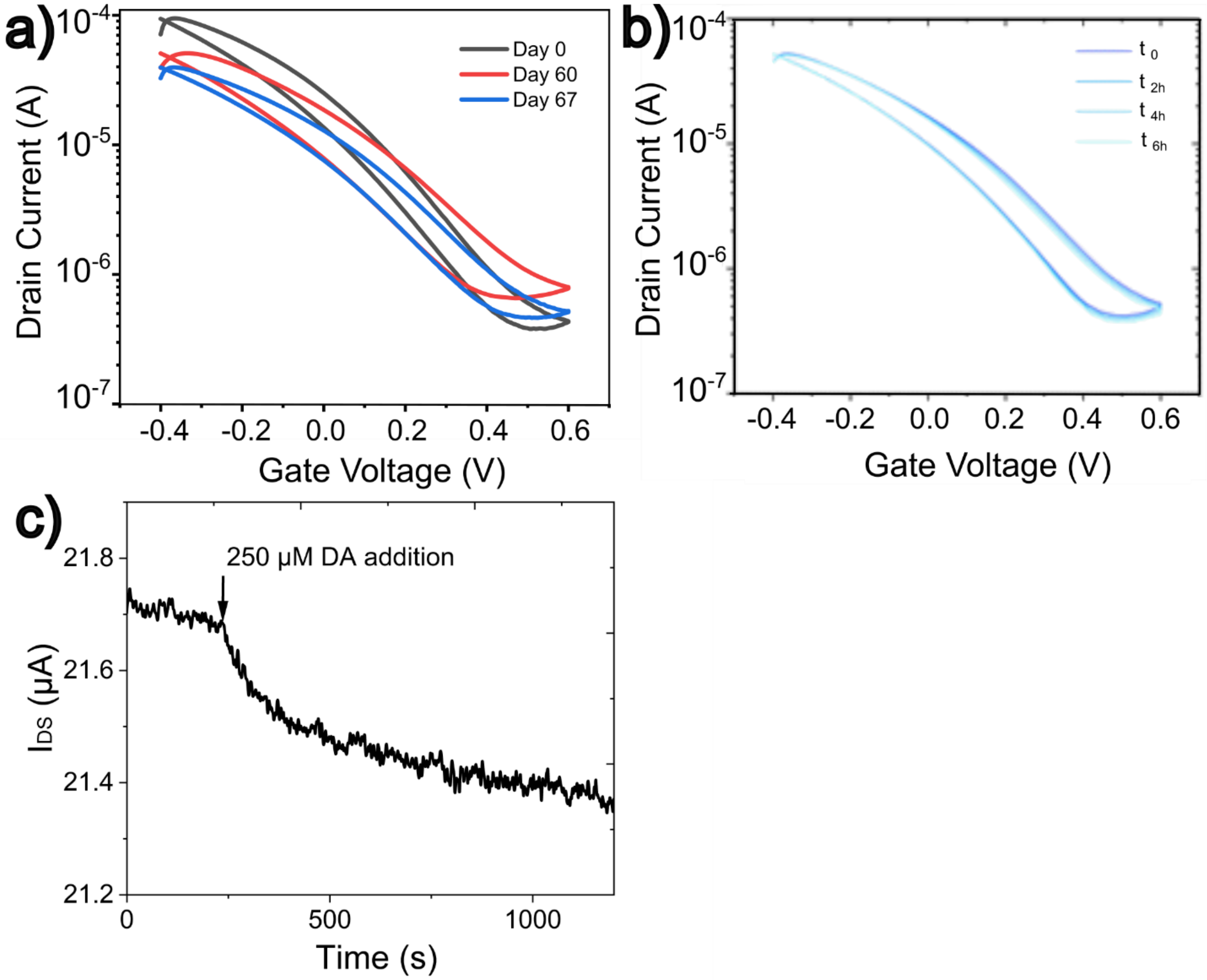

Fig. S9 a) Stability of the transfer curve of PEDOT-CQD OECT along multiple tests during 67 days and b) 6 hour-period time. c) Initial test of dopamine sensing using PEDOT-CQD OECT. The measurements were taken at constant drain voltage (V_d) of −0.4 V.

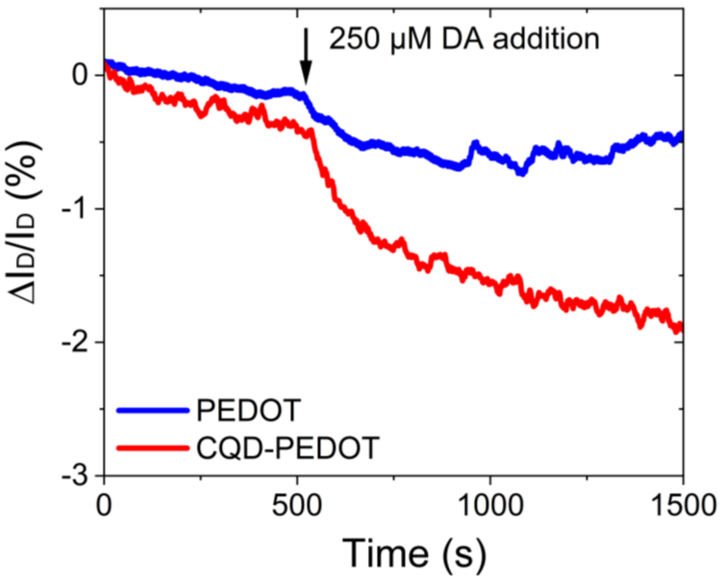

Fig. S10 Increased Sensitivity for DA Detection in PEDOT-Based and CQD-PEDOT-Based OECT Devices.

References

1. Christé S, Esteves da Silva JCG, Pinto da Silva L (2020) Evaluation of the Environmental Impact and Efficiency of N-Doping Strategies in the Synthesis of Carbon Dots Materials 13(3):504

2. Paulo-Mirasol S, Izquierdo C, Alemán C, Armelin E, Torras J (2023) Flexible electrode based on nitrogen carbon quantum dots for dopamine detection Appl Surf Sci 626:157241

3. Vercelli B, Donnini R, Ghezzi F, Sansonetti A, Giovanella U, La Ferla B (2021) Nitrogen-doped carbon quantum dots obtained hydrothermally from citric acid and urea: The role of the specific nitrogen centers in their electrochemical and optical responses Electrochim Acta 387:138557

4. Kumar SS, Mathiyarasu J, Phani KLN, Yegnaraman V (2006) Simultaneous determination of dopamine and ascorbic acid on poly (3,4-ethylenedioxythiophene) modified glassy carbon electrode J Solid State Electrochem 10(11):905-13

5. Gamboa J, Paulo-Mirasol S, Espona-Noguera A, Enshaei H, Ortiz S, Estrany F, et al. (2024) Biodegradable Conducting PVA-Hydrogel Based on Carbon Quantum Dots: Study of the Synergistic Effect of Additives J Polym Environ

6. Chen D, Wu W, Yuan Y, Zhou Y, Wan Z, Huang P (2016) Intense multi-state visible absorption and full-color luminescence of nitrogen-doped carbon quantum dots for blue-light-excitable solid-state-lighting J Mater Chem C 4(38):9027-35
